# Intestinal Obstructions

**Published:** 1885-01

**Authors:** 


**Intestinal Obstructions.** Their Varieties, with their Pathology, Diagnosis and Treatment. The Jacksonian Prize Essay of the Royal College of Surgeons of England, 1883. By FREDERICK TREEVES, F. R. C. S., Surgeon to and Lecturer on Anatomy at the London Hospital; Hunterian Professor of Anatomy at the Royal College of Surgeons, England. With sixty illustrations. Philadelphia: Henry C. Lea's Sons & Co. 1884.

The accomplished author of this work emphasizes the importance of the subject he selected for the Jacksonian Prize Essay, by stating that in England alone over two thousand individuals die annually from various forms of obstruction of the bowels, exclusive of hernia. His own experience in the fatality of this form of disease is corroborated by medical men of large practice. We are interested in the subject and the manner in which it is here treated, and we find in its perusal a mine of knowledge which we are sure will add greatly to the resources of the profession in treating these formidable diseases. The work is such a one as the general practitioner should have in his library.

